# Drivers of variation in occurrence, abundance, and behaviour of sharks on coral reefs

**DOI:** 10.1038/s41598-021-04024-x

**Published:** 2022-01-14

**Authors:** E. Lester, T. Langlois, I. Lindgren, M. Birt, T. Bond, D. McLean, B. Vaughan, T. H. Holmes, M. Meekan

**Affiliations:** 1 Australian Institute of Marine Science, The University of Western Australia, 35 Stirling Highway, Perth, WA 6009 Australia; 2 The University of Western Australia Oceans Institute, The University of Western Australia, 35 Stirling Highway, Perth, WA 6009 Australia; 3 The School of Biological Sciences, The University of Western Australia, 35 Stirling Highway, Perth, WA 6009 Australia; 4 Marine Science Program, Department of Biodiversity, Conservation and Attractions, 17 Dick Perry Avenue, Kensington, WA 6151 Australia

**Keywords:** Marine biology, Ecology

## Abstract

Quantifying the drivers of population size in reef sharks is critical for the development of appropriate conservation strategies. In north-west Australia, shark populations inhabit coral reefs that border growing centres of human population, industry, and tourism. However, we lack baseline data on reef sharks at large spatial scales (hundreds of km) that might enable managers to assess the status of shark populations in the face of future development in this region. Here, we examined the occurrence, abundance and behaviour of apex (*Galeocerdo cuvier, Carcharhinus plumbeus*) and reef (*C. amblyrhynchos, C. melanopterus, Triaenodon obesus*) sharks using > 1200 deployments of baited remote underwater stereo-video systems (stereo-BRUVs) across > 500 km of coastline. We found evidence for species-specific influences of habitat and fishing activities on the occurrence (probability of observation), abundance (MaxN) and behaviour of sharks (time of arrival to the stereo-BRUVs and likelihood of feeding). Although the presence of management zoning (No-take areas) made little difference to most species*, C. amblyrhynchos* were more common further from boat ramps (a proxy of recreational fishing pressure). Time of arrival for all species was also influenced by distance to boat ramp, although patterns varied among species. Our results demonstrate the capacity for behavioural metrics to complement existing measures of occurrence and abundance in assessing the potential impact of human activities on shark populations.

## Introduction

Sharks are top-order predators that play important roles in the shallow water ecosystems of reefs, embayments and estuaries throughout tropical and temperate oceans by transferring energy and predation risk across seascapes^1–5^. The coasts bordering these shallow habitats are often centres of human population and activities, notably tourism, recreational and commercial fishing, and industrial development (ports, mining facilities etc.). These anthropogenic pressures imperil the long-term persistence of elasmobranch populations, a problem compounded by the slow life histories of many species, increasing their vulnerability and hindering population recovery^6,7^.

**Figure 1:**
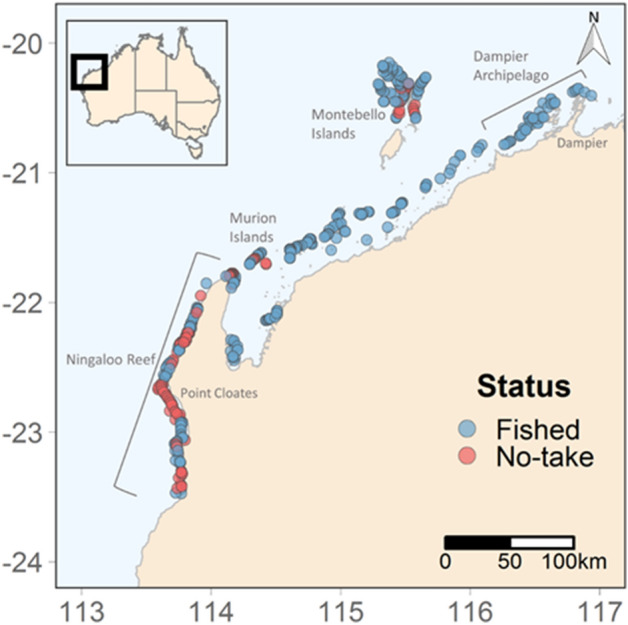
Map of BRUVs deployments in north-west Australia. Colour of circles indicates whether the BRUVs was deployed inside a No-Take Area (red) or in an area where fishing is permitted (Blue). Figure was generated using R^14^ using ggplot2^15^ and ggmap^16^.

In response to growing anthropogenic pressures, management agencies are establishing strategies for spatial management of marine resources using networks of marine reserves as a tool to conserve vulnerable communities and overall biodiversity^8^. To accommodate the needs of various stakeholders, management planning typically includes areas where fishing is excluded (No-Take Areas) and other areas where some types of extractive activities are restricted. Several studies have used BRUVs as a tool to evaluate the efficacy of marine reserves on shark abundance in both tropical and temperate reefs^9,10^. The extent to which such strategies effectively protect highly mobile species such as sharks is a subject of debate given the size of their ranges relative to the area of protection (Ward-Paige et al. 2012). There is, however, evidence that the abundance and biomass of predatory fishes is higher inside marine reserves and negatively correlated with human activities, such as coastal development, human population size, and cultivated land^11,12^. For sharks, recent modelling studies combining data from acoustic telemetry and baited remote underwater video systems (BRUVs) across 36 countries suggest that the median width of No-Take Areas in coral reef systems (9.4 km) would need to be increased by five times and be accompanied by strict enforcement in order to sufficiently protect populations of common reef-associated shark species, such as whitetip (*Triaenodon obesus*), blacktip (*Carcharhinus melanopterus*), and grey (*C. amblyrhynchos*) reef sharks^13^. Such models imply that in many situations, current systems of spatial management are unlikely to offer adequate protection to larger, more mobile components of the predatory community of reef sharks (Table 1, Fig. 1).

**Table 1:** Description and summary of the five variables used in analysis.

Variable	Description	Description of variable levels	Source
Status	Factor describing whether the stereo-BRUV was deployed inside a No-Take Zone or an area open to fishing	No-Take Zone: fishing prohibited (22.4% of data)	GlobalArchive
		Fished: fishing permitted (77.6%)	
Standard deviation of relief	The standard deviation of the height and structural complexity of the substrate	0–5	GlobalArchive
		0—Flat substrate, sandy, rubble with few features. ~ 0 substrate slope	
		1—Some relief features amongst mostly flat substrate/sand/rubble. < 45 degree substrate slope	
		2—Mostly relief features amongst some flat substrate or rubble. ~ 45 substrate slope	
		3—Good relief structure with some overhangs. > 45 substrate slope	
		4—High structural complexity, fissures and caves. Vertical wall. ~ 90 substrate slope	
		5—Very high structural complexity, numerous large holes and caves. Vertical wall. ~ 90 substrate slope	
		Range: 0–2.17	
		Mean: 0.41	
Reef cover	The total reef cover, which is the sum percentage cover of habitat that was classified as reef, sponges, ascidians and macroalgae	Range: 0–1	GlobalArchive
		Mean: 0.47	
Depth	Factor describing the depth of the stereo-BRUV deployment	Shallow: < 25 m (61.7% of data)	GlobalArchive
		Deep: > 25 m (38.3% of data)	
Distance to ramp (square root transformation)	The minimum Euclidean distance from the stereo-BRUV deployment to the closest boat ramp in metres	Range: 32.7–408 m	GlobalArchive
		Mean: 225.9 m

Besides management, a suite of other factors can drive the occurrence and abundance of sharks on coral reefs. These include the structural complexity of habitat^17^, life stage^18^, and isolation from human activities (e.g. fishing and boating)^7,19,20^. Although abundance is a widely used and standardized metric to assess the status of fish populations, behaviour is potentially a more sensitive measure of anthropogenic disturbance^21^. There is contrasting evidence surrounding the direction and magnitude of changes in behaviours of sharks driven by anthropogenic factors. For example, higher depredation rates by sharks on fishes caught by recreational anglers have been recorded in areas that are fished more regularly in Western Australia^22^. This suggests that recreational fishing may select for boldness or alter shark behaviour due to the availability of hooked fish as prey^23^. Conversely, evidence from New Caledonia suggested that grey reef sharks (*C. amblyrhynchos*) had increased wariness and were less likely to attempt to feed on bait in areas that were close to human population centres^20^. Determining the drivers of such differences in behaviour over reef-wide scales could enable the use of standardized behavioural metrics that might provide more comprehensive insights into the impacts of human activities on the shark assemblages of coral reefs.

Baited Remote Underwater Stereo-Video Systems (stereo-BRUVs) are commonly used to assess the abundance of sharks in coral reefs^24^. This method can also provide an opportunity to examine the influence of human activities (or isolation from humans) on the behaviour of sharks recorded in the field of view of the cameras^20^. Recently, behavioural metrics such as time to arrival (time taken for first individual of a species to appear in the field of view of stereo-BRUVs) and number of interactions with a bait bag have been used to examine interactions between predatory species and their prey^25–29^. These studies hypothesized that prey would take longer to arrive in the field of view of stereo-BRUVs in less structurally complex habitats that offer little refuge from predators^27^ or are in areas with exposure to sharks^28^. Additionally, rays are less likely to forage from the bait bag in areas with higher predator exposure^25–29^. This framework can be expanded to investigate the effect of proximity to human activities on the behaviour of sharks on coral reefs. For example, if human activities are perceived as threatening or disturbing, time to arrival by sharks at a BRUVs would increase and likelihood to feed from a bait bag would decrease. Conversely, if human activities provided a benefit (i.e. feeding opportunities) time to arrival would decrease and likelihood to feed would increase.

In the north-west of Western Australia, relatively intact populations of sharks inhabit coral reefs adjacent to infrastructure (e.g. ports, loading facilities) that support mineral extraction (principally petrochemical and iron ore) industries. The development of these facilities has been accompanied by growth in coastal centres of human populations and associated activities (https://www.abs.gov.au/census). Additionally, other parts of this north-west coast have become hubs for tourism and recreational fishing^30^. In 2017/2018, the boat-based recreational fishing catch across Western Australia included over 1.32 million individual finfish (49% released)^30^. Most of this fishing effort occurred in the West Coast bioregion surrounding Perth (74% of recreational fishing effort), but the Gascoyne Coast (including the Ningaloo Reef) had the second-highest level of recreational fishing effort (12%), followed by the North Coast (8%)^30^. Given the potential for these anthropogenic activities to adversely impact shark populations, it is important to develop regional benchmarks of the status of shark populations to ensure appropriate conservation and management strategies. This is given added impetus due to north-west Australia providing a refuge for species extirpated from parts of their former range, such as the Coral Triangle^7^.

Here, we use > 1200 deployments of stereo-BRUVs to investigate the occurrence, abundance, and behaviour (time to arrival) of sharks across more than 500 km of the north-west coast of Western Australia Fig. 1. A full-subsets information theoretic approach to the analysis of this data set allowed us to investigate the relative importance of environmental (habitat complexity, depth) and anthropogenic (management and distance from boat ramp) factors as drivers of variation in these metrics Table 1. This statistical approach is widely used for large-scale analyses in complex systems (e.g.^31,32^), as it facilitates the examination of patterns that may be driven by multiple correlated variables. We hypothesised that the occurrence and abundance of reef sharks would be higher further from anthropogenic activities and in areas of greater habitat complexity. Additionally, we hypothesized that sharks would arrive in the field of view of the stereo-BRUVs faster and be more likely to feed at increasing distances from human activities.

## Results

### Shark assemblages

A total of 889 (sum MaxN) sharks comprising 19 different species were observed across the 1273 stereo-BRUV deployments. The MaxN of the five most abundant sharks (*C. amblyrhynchos, T. obesus*, *C. melanopterus*, *G. cuvier*, and *C. plumbeus)* per deployment ranged from 0 to 6 with a mean of 0.7 (SE 0.34) reef sharks (*C. amblyrhynchos*, *C. melanopterus*, and *T. obesus*) and 0.04 (SD 0.17) apex sharks (*G. cuvier* and *C. plumbeus*) recorded per 60 min deployment. In all deployments, the most abundant apex shark was *G. cuvier* (sum MaxN = 60) followed by *C. plumbeus* (sum MaxN = 44). In deployments in depths of < 25 m (n = 897), the most abundant reef shark species were *C. amblyrhynchos* (sum MaxN = 199), *C. melanopterus* (sum MaxN = 84), and *T. obesus* (sum MaxN = 62).

For the five most abundant sharks across all 1273 BRUV deployments, the time of arrival per deployment ranged from 3 to 59 min (mean = 28 min 30 s SD = 4 min 42 s). For apex sharks, the mean time of arrival was 26 min 40 s (SD = 16 min 32 s). For reef sharks on deployments < 25 m depth, the mean time of arrival was 23 min 14 s (SD = 14 min 27 s) for *C. amblyrhynchos*, 27 min 32 s (SD = 16 min 57 s) for *C. melanopterus*, and 20 min 26 s (SD = 13 min 17 s) for *T. obesus*.

### Apex sharks

**Figure 2:**
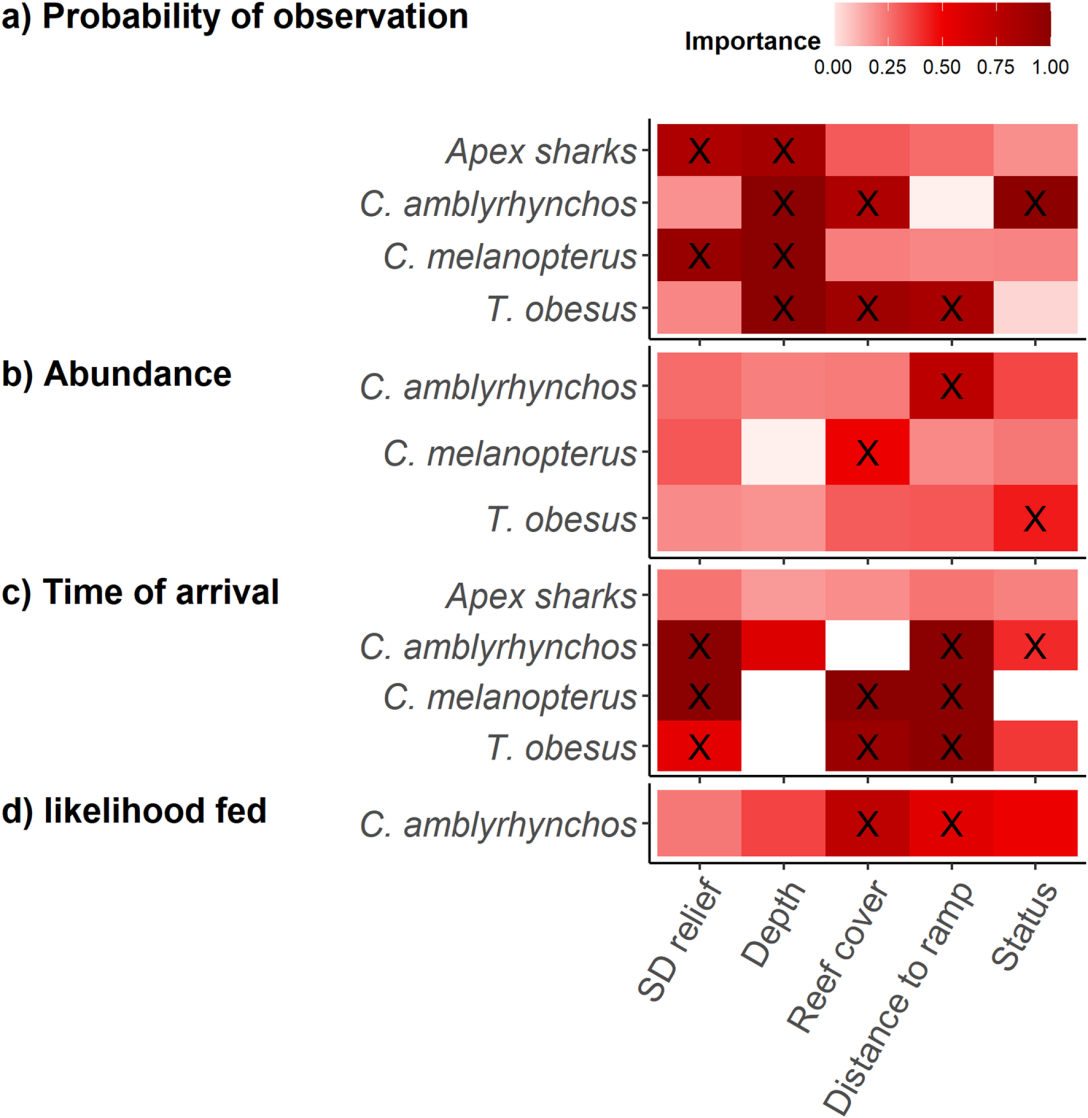
Importance scores based on summed AIC weights from full subsets analysis exploring the influence of five variables on (**a**) probability of occurrence, (**b**) abundance and (**c**) time of arrival on BRUVs, and (d) likelihood fedfor each shark taxa. The ‘X’ symbols indicate variables that were included in the most parsimonious models (See Table 1). Figure was generated using R^14^ using ggplot2^15^.

**Figure 3:**
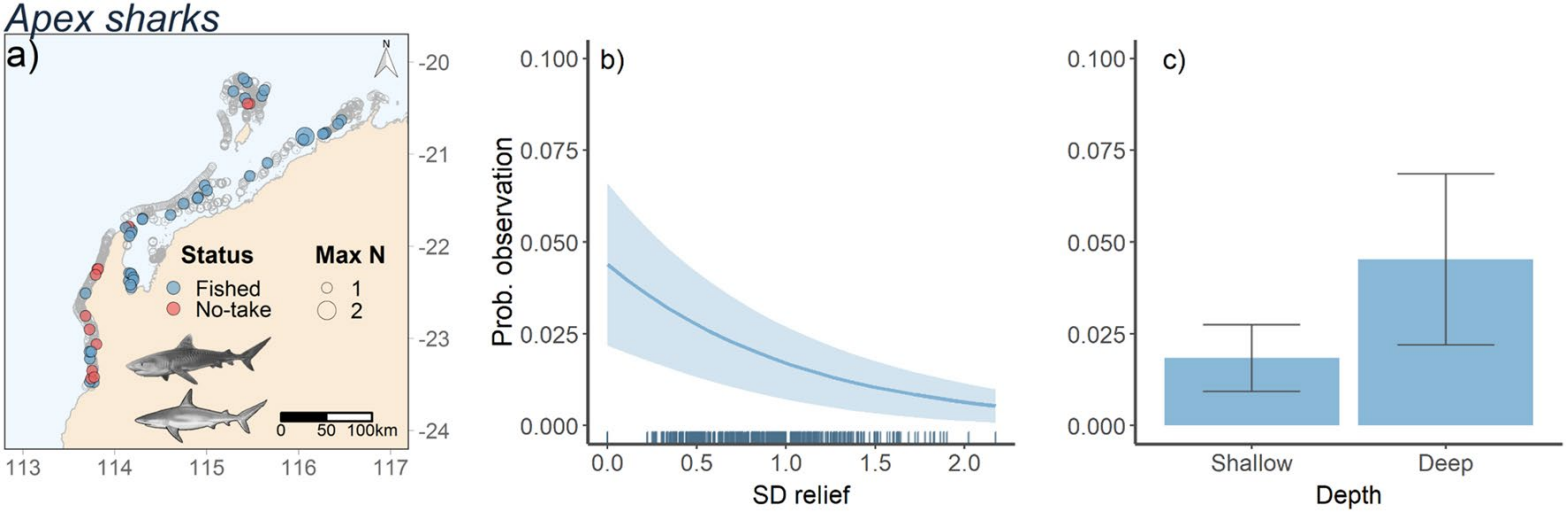
Map of stereo-BRUVS deployments in north-west Australia (indicated by grey open circles. Colour of circles indicates whether the stereo-BRUVS was deployed inside a No-Take Area (red) or in an area where fishing is permitted (Blue). Size of the circle shows the MaxN of apex sharks (*Galeocerdo cuvier, Carcharhinus plumbeus*) in each deployment (**a**). Predicted apex shark probability of occurrence (**a**,**b**) as functions of variables present in the most parsimonious models (Table 2) from full-subsets GAMM analysis. Ribbons and error bars represent 95% confidence intervals. Figure was generated using R^14^ using ggplot2^15^ and ggmap^16^.

The most parsimonious model for the occurrence of an apex shark included standard deviation of relief and depth (Table 2, Fig. 2a). The occurrence of apex sharks was negatively correlated with standard deviation of relief and increased at depths greater than 25 m (Fig. 3a-c). As a MaxN greater than one was only observed once, it was not possible to run presence only models. Analysis for time of arrival generated support for the null model (Table 2). There were insufficient observations of feeding behaviour to model the likelihood that apex sharks fed from the bait bag.

**Table 2:** Top Generalised Additive Mixed Models (GAMMs) for predicting probability of observation, abundance given observation and time of arrival from full-subsets analyses for the five shark groups. The difference between the lowest reported Akaike Information Criterion corrected for small sample size (AICc), AICc weights (wAICc), variance explained (R^2^) and estimated degrees of freedom (EDF) are reported for model comparison. The most parsimonious model is shown in bold and was defined as the model that contains the fewest variables and the lowest EDF within two units of the lowest AICc.

Shark group	Model	ΔAICc	ωAICc	R^2^	EDF
**Probability of occurrence**					
Apex sharks	SD relief + sqrt distance to ramp + depth	0	0.23	0.02	12.71
	**SD relief + depth**	0.19	0.21	0.02	11.92
	SD relief + reef cover	1.16	0.13	0.02	12.52
	SD relief + sqrt distance to ramp + reef cover	1.57	0.1	0.02	14.3
*C. amblyrhynchos*	**Reef cover + sqrt depth + status**	0	0.81	0.15	7.47
*C. melanopterus*	SD relief + sqrt depth	0	0.36	0.03	5.85
	SD relief + sqrt depth + status	1.19	0.19	0.03	6.85
	SD relief + reef + sqrt depth	1.25	0.19	0.03	7.03
	SD relief + sqrt distance to ramp + sqrt depth	1.29	0.18	0.03	6.85
*T. obesus*	**Reef cover + sqrt distance to ramp + sqrt depth**	0	0.74	0.04	8.24
**Abundance**					
*C. melanopterus*	**Reef cover**	0	0.27	0.11	4.92
	SD relief + reef cover	0.27	0.24	0.14	6.55
	Reef cover + status	1.29	0.14	0.11	5.91
	Sqrt distance to ramp + reef cover	1.87	0.11	0.12	6.41
*C. amblyrhynchos*	**Sqrt distance to ramp**	0	0.18	0.04	4.69
	Sqrt distance to ramp + status	0.61	0.13	0.05	5.74
	SD relief + sqrt distance to ramp	1.71	0.07	0.04	5.71
*T. obesus*	**Status**	0	0.14	0.06	4
	Sqrt distance to ramp	1.03	0.09	0.08	4
	Reef cover + status	1.21	0.08	0.09	5.38
	Reef cover	1.4	0.07	0.10	4.53
	Sqrt distance to ramp + status	1.54	0.07	0.09	5.43
	SD relief + status	1.98	0.05	0.07	5
**Time of arrival**					
Apex sharks	**SD relief + sqrt distance to ramp**	0	0.31	0.11	6.43
	SD relief + reef cover + sqrt distance to ramp	0.65	0.22	0.13	8
	SD relief + sqrt distance to ramp + depth	1.23	0.16	0.12	7.36
	SD relief + sqrt distance to ramp + status	1.77	0.12	0.11	7.41
*C. melanopterus*	**SD relief + reef cover + sqrt distance to ramp**	0	0.99	0.14	7.43
*C. amblyrhynchos*	SD relief + sqrt depth + sqrt distance to ramp	0	0.59	0.10	8.65
	**SD relief + sqrt distance to ramp + status**	0.73	0.41	0.10	7.93
*T. obesus*	**SD relief + reef + sqrt distance to ramp**	0	0.47	0.10	6.98
	Reef cover + sqrt distance to ramp + status	0.48	0.37	0.11	7.56
**Likelihood fed**					
*C. amblyrhynchos*	Reef cover + sqrt distance to ramp + status	0	0.14	0.04	6.82
	**Reef cover + sqrt distance to ramp**	**0.67**	**0.1**	**0.04**	**5.82**
	Reef cover + sqrt depth + sqrt distance to ramp	1	0.08	0.03	7.53
	Reef cover + sqrt depth + status	1.09	0.08	0.03	7.5

### Carcharhinus amblyrhynchos

**Figure 4:**
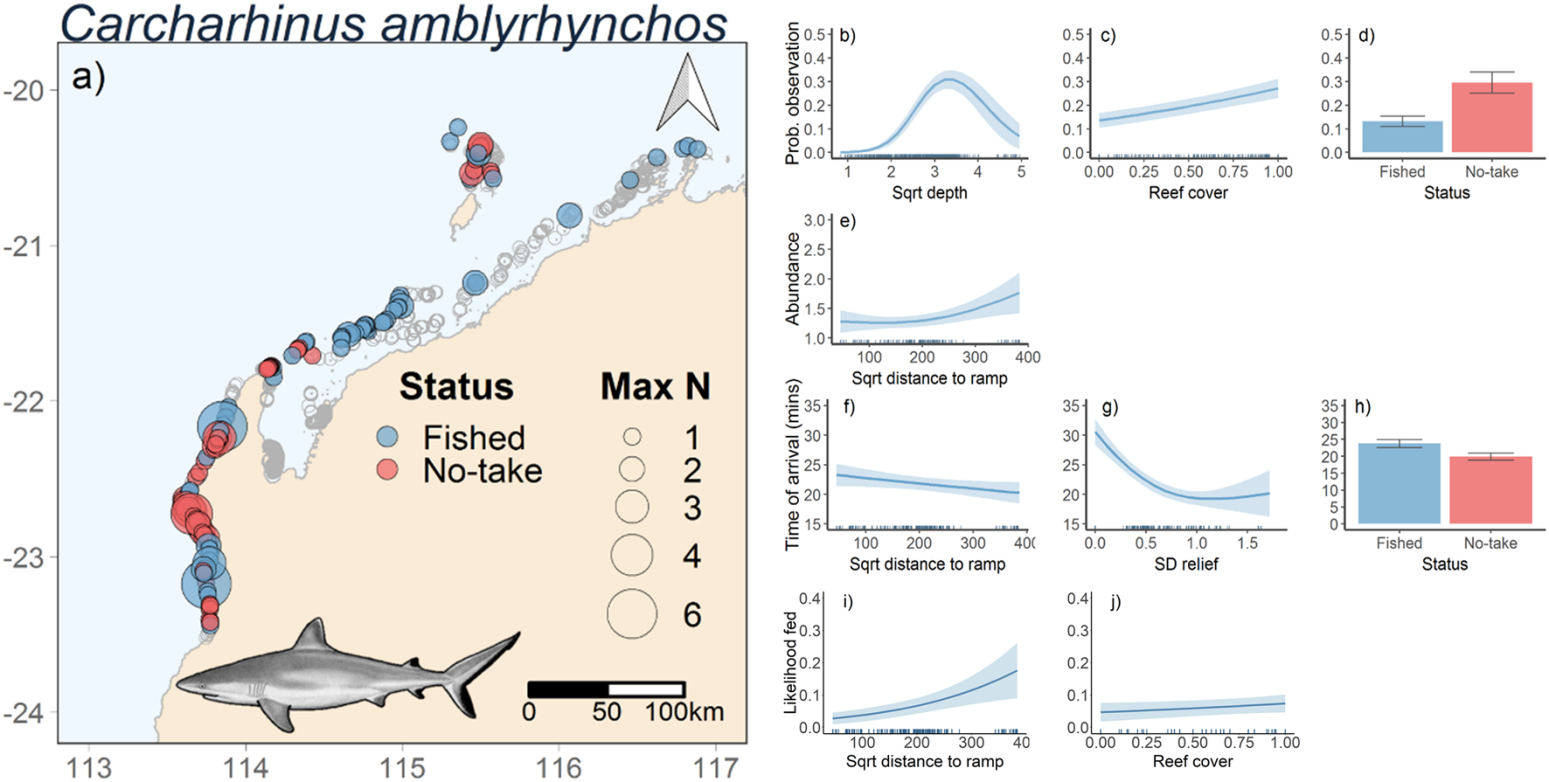
Map of stereo-BRUVS deployments in north-west Australia (indicated by grey open circles. Colour of circles indicates whether the stereo-BRUVS was deployed inside a No-Take Area (red) or in an area where fishing is permitted (Blue). Size of the circle shows the MaxN of *Carcharhinus amblyrhynchos* in each deployment (**a**). Predicted *C. amblyrhynchos* probability of occurrence (**b**,**c**,**d**), abundance (MaxN; **e**), time of arrival on stereo-BRUVS (**f**,**g**,**h**), and likelihood fed (**i**, **j**) as functions of variables present in the most parsimonious models (Table 2) from full-subsets GAMM analysis. Ribbons and error bars represent 95% confidence intervals. Figure was generated using R^14^ using ggplot2^15^ and ggmap^16^.

For *C. amblyrhynchos*, the most parsimonious model for occurrence included depth, reef cover, and status (Table 2, Fig. 2a). The occurrence of *C. amblyrhynchos* was highest at depths of 9–20 m, was positively correlated with reef cover, and was higher inside No-Take Areas compared to fished areas (Fig. 4a–d). The abundance (MaxN > 1) of *C. amblyrhynchos* was positively correlated with distance from the boat ramp (Fig. 4e). The most parsimonious model for the time to arrival included distance from the nearest boat ramp (m), standard deviation of relief and status (Table 2, Fig. 2c). The time of arrival for *C. amblyrhynchos* was negatively correlated with distance from boat ramp and standard deviation of relief, and was lower in No-Take Zones compared to fished areas (Fig. 4f–h). Similarly, likelihood to feed was positively correlated with distance to boat ramp and weakly correlated with the level of reef cover (Fig. 4i–j). Importance scores also indicated support for status as a predictor variable (Fig. 2d).

### Carcharhinus melanopterus

**Figure 5:**
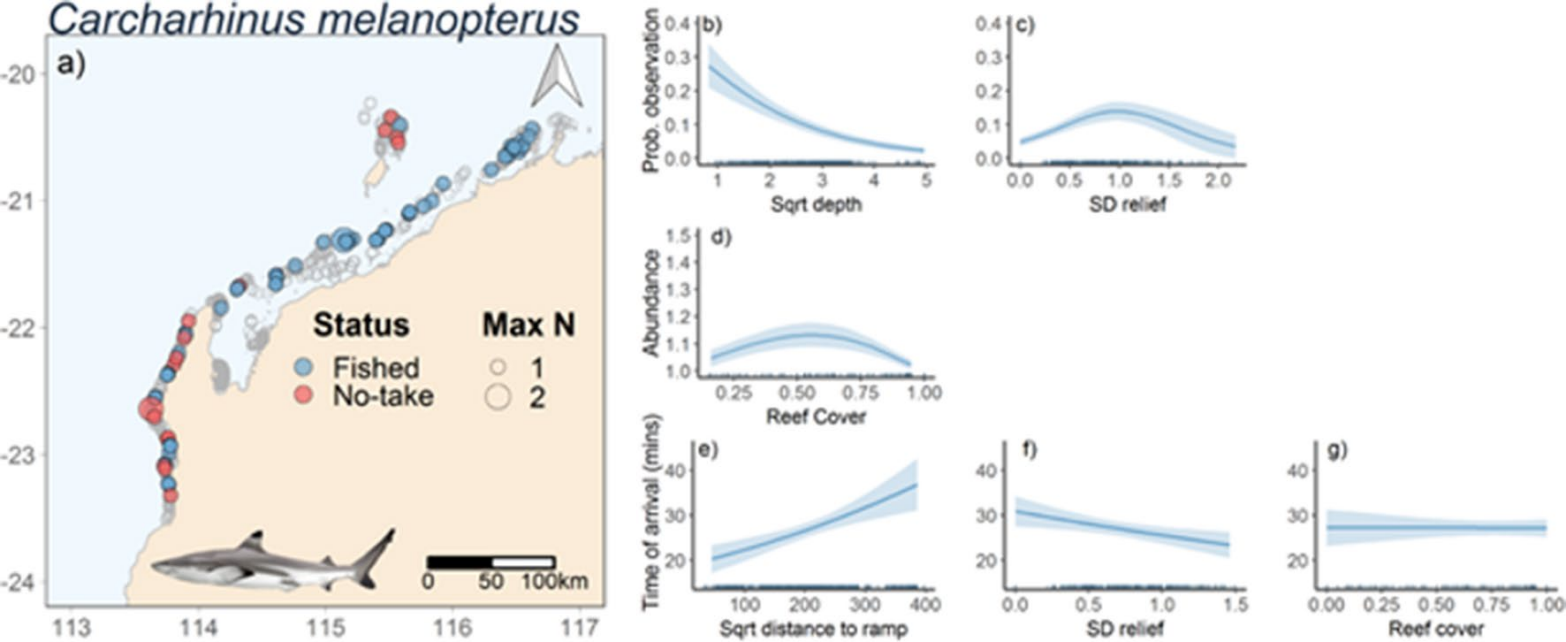
Map of stereo-BRUVS deployments in north-west Australia (indicated by grey open circles. Colour of circles indicates whether the stereo-BRUVS was deployed inside a No-Take Area (red) or in an area where fishing is permitted (Blue). Size of the circle shows the MaxN of *Carcharhinus melanopterus* in each deployment (**a**). Predicted *C. melanopterus* probability of occurrence (**b**,**c**), abundance (MaxN; **d**) and time of arrival on BRUVs (**e**,**f**,**g**) as functions of variables present in the most parsimonious models (Table 2) from full-subsets GAMM analysis. Ribbons and error bars represent 95% confidence intervals. Figure was generated using R^14^ using ggplot2^15^ and ggmap^16^.

Occurrence of *C. melanopterus* was negatively correlated with depth and was highest at intermediate levels of relief (Fig. 5a–c). There was a weak trend of increasing presence only abundance at intermediate levels of reef cover (Fig. 5d). The most parsimonious model for time to arrival of *C. melanopterus* included standard deviation of relief, reef cover, and distance to boat ramp (Table 2, Fig. 2c). Time to arrival was positively correlated with distance to boat ramp, negatively correlated with standard deviation of relief, and had no discernible trend was identified for reef cover (Fig. 5e–g). *Carcharhinus melanopterus* did not feed from the bait bag, which prevented an analysis for likelihood to feed from the bait bag.

### Triaenodon obesus

**Figure 6:**
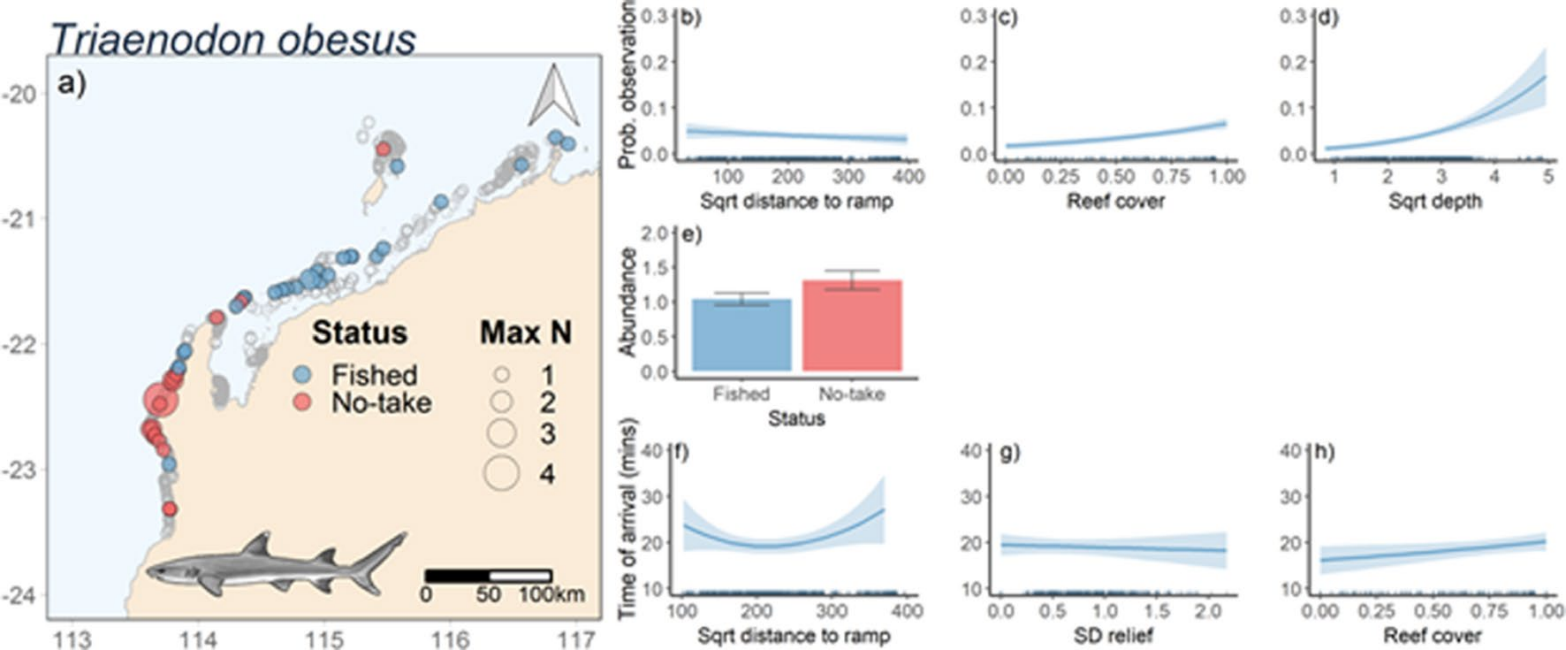
Map of stereo-BRUVS deployments in north-west Australia (indicated by grey open circles. Colour of circles indicates whether the stereo-BRUVS was deployed inside a No-Take Area (red) or in an area where fishing is permitted (Blue). Size of the circle shows the MaxN of *Triaenodon obesus* in each deployment (**a**). Predicted *T. obesus* probability of occurrence (**b**,**c**,**d**), abundance (MaxN; **e**) and time of arrival on BRUVs (**f**,**g**,**h**) as functions of variables present in the most parsimonious models (Table 2) from full-subsets GAMM analysis. Ribbons and error bars represent 95% confidence intervals. Figure was generated using R^14^ using ggplot2^15^ and ggmap^16^.

The occurrence of *T. obesus* was negatively correlated with distance from the nearest boat ramp and positively correlated with reef cover and depth (Fig. 6a–d). For abundance of *T. obesus,* the most parsimonious model for abundance included status (Fig. 2b) and presence only abundance was highest in No-Take Areas (Table 2, Fig. 6e). Time of arrival for *T. obesus* was best predicted by standard deviation of relief, reef cover and distance to the closest boat ramp (Table 2, Fig. 2c). Time of arrival was quickest at intermediate distances from boat ramps, negatively correlated with standard deviation of relief and positively correlated with reef cover (Fig. 6f–h). There was not sufficient feeding behaviour observations to model likelihood to feed for *T. obesus*.

## Discussion

Our study found evidence for species-specific influences of habitat and human activities on the occurrence, abundance and behaviour of shark species across more than 500 km of coastline in north-west Australia. In terms of occurrence (probability of observation), abundance (MaxN), and behaviour (time of arrival to stereo-BRUVs and likelihood of feeding), the zoning status of the reef made little difference to apex sharks and *C. melanopterus*, although occurrence of *C. amblyrhynchos* and abundance of *T. obesus* was higher inside No-Take Areas. In contrast, larger distances to the nearest boat ramp (a proxy of human disturbance) resulted in shorter arrival times and higher likelihood of feeding for the most common species, *C. amblyrhynchos*. Although shorter distances to the nearest boat ramp resulted in shorter arrival times of *C. melanopterus* and *T. obesus*, these species did not feed frequently enough from the bait bag to model likelihood of feeding. Despite sampling over an extensive spatial range and recording a high number of sharks, our models explained a very small amount of variation (see Table 2), demonstrating the complex nature of the associations between large mobile species and habitat, management and anthropogenic activities over large spatial scales.

In a global context, our observation of 0.7 reef sharks per hour and 0.04 apex sharks per hour was similar to other comparable protected reef systems, such as Fiji (0.8 reef sharks per hour^33^), and the Rowley Shoals (0.65 reef sharks per hour^24^) and northern Great Barrier Reef (0.05 *G. cuvier* per hour^7^). However, our estimate of shark abundance was lower than remote reefs in New Caledonia (> 2.5 reef sharks per hour^34^), Cocos Keeling Islands (~ 2 *C. amblyrhynchos* per hour^35^), and Minerva Reef in Tonga (0.2 *G. cuvier* per hour^7^). Such differences in average numbers of sharks recorded by BRUVs might be due to a number of potential sources, such as variation in reef productivity among locations^35^, or events that attract and aggregate these predators in reef waters, such as turtle nesting^36^ in the case of apex predators, or spawning schools of fishes for reef sharks^37^. Repeated series of BRUVs sampling will be required to determine if these differences are fixed in nature or due to ephemeral events.

We found small increases in the occurrence of *C. amblyrhynchos* and the abundance of *T.obesus* inside No-Take Areas. This result was somewhat surprising given there is generally a lack of targeted shark fishing from commercial^38^ and recreational fishers. The weak effect of management zones on shark abundance may be in part be explained by the geography of the region, as the reef is close to shore and there is a high degree of connectivity among No-Take Areas. In comparison, studies that have found large effects of management zones on shark abundance usually make comparisons between offshore reefs that are difficult to access or between protected versus unprotected coral reef atolls where deep water prevents shark movements between atolls^10,24^. Our results from coastal north-west Australia suggest that No-Take Areas may provide some benefits to reef sharks despite these zones being established to conserve a broad representation of the region’s marine habitats, flora, and fauna rather than being implemented to specifically target shark populations^39^. These patterns may be due to increased prey availability inside No-Take Areas^33^ or to these No-Take Areas encompassing favourable habitat for sharks^40^. However, a recent analysis of movement patterns of sharks suggests that to protect at least 50% of all local *T. obesus*, which are the most site-attached species in our study, No-Take Areas would need to encompass at least ~ 10 km of continuous reef habitat^13^. The largest No-Take Area in the region, Point Cloates, encompasses ~ 24 km of continuous reef suggesting it is sufficient to protect 50% of local *T. obesus* and *C. melanopterus* populations, but the reserve would need to be expanded to include 30 km of continuous reef to achieve this same target for *C. amblyrhynchos*^13^.

In contrast, we found no impact of spatial management of fishing activity on apex sharks, which is unsurprising given that these species show a much greater degree of mobility than reef sharks and have home ranges that often traverse the boundaries of entire marine parks. At Ningaloo, satellite tags deployed on tiger sharks revealed periods of residency that were interspersed with large scale movements (100–1000 s km), with individuals sometimes travelling to Indonesia and the temperate waters in south-west Australia^41^. For these species, local management is unlikely to mitigate the threats faced in the open ocean such as longline fishing. However, modelling approaches have demonstrated that No-Take Areas can have positive effects on numbers of large, mobile sharks, such as *G. cuvier*, if they encompass highly suitable habitats for these species^68,69^. Such benefits will depend upon the amount of time individual sharks spend within the boundaries and the life-history stages that experience this protection. In north-west Australia, *G. cuvier* may display periods of residency near the coast lasting several months^41^, so it is possible that No-Take Areas might protect the species during this time.

Distance to a boat ramp influenced the occurrence, abundance, and behaviour of reef sharks. We found that *C. amblyrhynchos* arrived faster to stereo-BRUVs and were more likely to feed on the bait bag at increasing distances from human activities. There are two potential (and non-exclusive) explanations for these relationships. Firstly, time to arrival at a BRUVs is highly correlated with abundance in both sharks and other predatory reef fishes^26,42,43^. Thus, higher abundances of *C. amblyrhynchos* at increasing distances from boat ramps might result in the observed decline in arrival times. Alternatively, human activities could be increasing the wariness, and therefore arrival times, of this species close to boat ramps, as was the case in New Caledonia^20^.

In contrast to *C. amblrhynchos*, C*. melanopterus* and *T. obesus* arrived sooner to the stereo-BRUVs closer to boat ramps. This result was not correlated with the abundance of these species. Although distance from human activities (e.g. human population density) or distance to boat ramp have been used in studies investigating patterns of shark abundance and behaviour (e.g.^19,20^), it is possible that these metrics are a coarse measure of human activity in north-west Australia. For example, distance to boat ramp may be correlated with another, unmeasured variable that is driving these patterns. This may be the case in our study, as C*. melanopterus* and *T. obesus* are more likely to inhabit reef lagoons and crests, which generally occur closer to boat ramps. For this reason, a more direct measure of human activities could provide an insight into the potential drivers of these patterns. For example, at Ningaloo there is evidence of behavioural links between reef sharks and the presence of recreational and charter fishers. From July 2015 to May 2016^22^, surveyed 248 fishing boats at boat ramps on the Ningaloo Reef coast and 155 boats at ramps on Exmouth Gulf. They found that fishers reported sharks removing fish from lines on almost 40% of trips in both locations. Importantly, there was marked spatial variation in depredation, with higher rates occurring in areas where there was greater fishing pressure. This suggests that sharks may display learned behaviour in response to the opportunity to scavenge a relatively easy meal, but this behaviour may be restricted to locations where fishing was more common. An experimental approach would be required to investigate any associations between fishing and the behaviour of reef sharks (see^23^).

Depth and habitat complexity (reef cover or standard deviation of relief) were important predictors of occurrence of all species of reef shark. These sharks were more likely to be observed in shallow depths (< 25 m), but within this range we found evidence of species-specific depth preferences. *C. melanopterus* were most likely to be observed in the shallowest deployments (0–4 m), and this species is known to inhabit complex habitats on reef flats within shallow lagoons^44–46^. *C. amblyrhynchos* were found at intermediate depths (4–9 m), whereas *T. obesus* were most likely to be observed in deeper deployments (16–25 m). This result for the latter species is unsurprising, given that it favours structurally complex reef edge and back reef habitats as they often consume prey that shelter in crevices and holes^45^. In addition to depth, reef cover was an important driver of occurrence of *T. obesus* and *C. amblyrhynchos*. Our finding that *C. melanopterus* was most abundant at intermediate levels of relief may be confounded by the shallow water depths in which it occurred, with the species more difficult to observe in complex reefs that could obscure parts of the field of view of the cameras. Time of arrival for all three species was quickest in areas of high structural complexity, which adds further support for suggestions that reef sharks prefer structurally complex habitat^17,35,45,47^. Such habitats may host a greater number and diversity of potential prey items as well as providing more refuge from the larger apex sharks that might prey on reef species^48,49^. If this is the case, environmental degradation and disturbance events that reduce the structural complexity of reef habitats may adversely affect reef sharks.

We recorded few apex sharks despite an intense sampling effort across a large spatial scale that included a broad variety of reef habitats. For *C. plumbeus* and *G. cuvier,* the two most abundant species, we found a trend of higher probability of occurrence in deeper (> 25 m) waters, and in areas of less structurally complex habitat. This is expected given that *G. cuvier* often inhabit open ocean waters^41^ and, these apex sharks have little need for shelter from other predators (at least at adult sizes). Tagging studies have also demonstrated that tiger sharks frequently visit shallow sandflat environments, which are likely to be important foraging areas for these predators^50,51^. By not incorporating this key habitat into our sampling, we may have underestimated the abundance of these apex predators in north-west Australia. Furthermore, stereo-BRUVS are biased towards sampling smaller species of shark, compared to other methods, such as longline surveys, which select larger species due to catchability and size selectivity biases^52,53^. This may also contribute to the low number of larger sharks recorded in our study. Although the low number of these species recorded on stereo-BRUVs may trigger concerns regarding the impact of historical commercial fishing activities in this region, (shark fisheries in the north-west closed in 2009) recent evidence from > 25 years of scientific longline and dropline surveys found fluctuating but stable patterns in catch rates and body size of these species^38^. This similarity in catch and size patterns before and after the closure of commercial fishing activities suggests that these large-bodied, apex sharks have always been a rare part of the assemblage in the region.

Our study examined patterns in the abundance and behaviour of sharks during the day, but it is important to note that habitat use^54^, activity levels^44,55^, and depth distributions^56^ of sharks can alter at night. Despite this sampling bias, our study provided a comprehensive survey of patterns in the distribution and abundance of sharks across hundreds of kilometres of tropical and sub-tropical reef. Sampling at night using stereo-BRUVs is a challenge for any study as it generally necessitates the use of lights that might alter the behaviour of both prey and predator species in the vicinity of the stereo-BRUVs. One solution to this problem might be to use biologging to examine temporal variation in shark behaviour and fine-scale movement patterns. This approach has demonstrated distinct diel patterns of activity for six co-occurring species of sharks in the Gulf of Mexico and provided evidence of strong temporal partitioning of foraging times^57^. Such data could aid the interpretation of stereo-BRUVs data sets.

Our results provide evidence that reef sharks have a strong affinity with structurally complex habitat with high levels of coral cover. We also found a small effect of distance from boat ramps and spatial patterns of management on the abundance and behaviour of reef sharks. Both results are difficult to interpret as distance from boat ramps is likely a very coarse measure of human disturbance in this region, whereas in terms of the impact of spatial management, our outcomes could be explained by a variety of equally plausible hypotheses. It might be that fishing pressure on sharks is too limited at this location to create distinct spatial patterns in shark abundance with No-Take Areas. Indeed, levels of commercial shark fishing in this region are low^58^. Alternatively, the current size of No-Take Areas may be inadequate to protect wider ranging sharks due to the large home ranges these species tend to occupy. Distinguishing among these alternatives will require detailed studies of the behaviour of both fishers and sharks in relation to spatial management strategies. We found species-specific drivers of reef shark occurrence, abundance and behaviour, which illustrates the value of pooling large data sets collected by multiple institutions for analysis. Our results also demonstrate the capacity for behavioural metrics to complement existing measures of abundance and occurrence in assessing habitat preferences and the potential impact of human activities on reef shark populations.

## Materials and methods

### Study area

Sampling occurred on shallow reefs on the continental shelf of north-west Australia from the southern point of the Ningaloo Reef to the Dampier Archipelago, where hard reef is considered a major component of the benthic community (Fig. 1). This area incorporates the Ningaloo, Montebello Islands and Barrow Islands marine parks and the Muiron Islands and Barrow Island marine management areas, as well as significant areas of coastal waters and islands without spatial management. Together the marine reserves form a network of 24 No-Take Areas encompassing an area of 123,089 hectares where boat based fishing is prohibited. No-Take Areas across the reserves vary greatly in size, ranging from 8 hectares to 44,752 hectares. It should be noted that coastal fishing is permitted in a subset of the No-Take Areas within the Ningaloo Marine Park.

### Sampling protocol and equipment

Surveys were completed between April 2014 and September 2015 using stereo-BRUVs deployed over 11 separate studies (Table S1). A total of 1273 deployments were conducted in 0.7–100 m of water (mean = 29.4 m, ± SD = 25.2 m) across a range of habitat types.

Stereo-BRUVs consisted of two high-definition video cameras (either Canon Legria HFG25 or GoPro Hero 3+) attached to a galvanised steel bar framed in a trapezium prism. Cameras are inwardly converged at 7 degrees, which provided an overlapping field of view. This configuration allowed for accurate measures of size and distance. Further information on configuration and calibration is available in^70^.

Each stereo-BRUV was baited with approximately 1 kg of commercially purchased frozen crushed pilchards (*Sardinops* spp.), which were contained within a plastic-coated wire mesh basket, attached to a conduit rod and positioned 1.2 m in front of the cameras. Each system was deployed by boat and left to film remotely for at least 60 min on the seafloor before being retrieved and re-deployed elsewhere. Neighbouring deployments were separated by at least 400 m to reduce the likelihood of fish swimming between stereo-BRUVs^59^.

### Video analysis

Stereo-BRUVs were analysed using the program EventMeasure (http://www.seagis.com.au). In each video, any shark present in the field of view was identified to species level. The time (minutes) difference between the BRUV landing on the seafloor and the first individual of each species of shark entering into the field of view was also recorded and hereinafter referred to as “time of arrival”. Additionally, we recorded if each species fed from the bait bag (1 = species fed, 0 = species did not feed), and was used to calculate likelihood fed. Relative abundance counts were obtained as the maximum number of individuals of a single shark species present in the field of view of each stereo-BRUV at one time (MaxN^42^). The stereo-configuration of the video systems allowed us to obtain accurate and precise measurements of shark length using EventMeasure. This stereo-video configuration also allowed for measures of distance, which was used to finalise measures of relative abundance by confirming whether individuals were within a sample boundary of 10 m from the cameras. Data checking and formatting of EventMeasure MaxN outputs used R language for statistical computing (R Core Team 2020) and R scripts provided in Langlois et al.^59^.

### Habitat variables

Measures of habitat and relief were obtained following protocols outlined in^72^. A 5 × 4 grid was overlaid on an image obtained from each stereo-BRUVS deployment. Within each grid rectangle, the dominant habitat type was characterised using eight broad categories from the CATAMI classification scheme: (1) hard corals, (2) macroalgae, (3) reef (boulders or pavement—including those covered in turfing algae + hard corals + macroalgae), (4) sand/rubble, (5) seagrass, (6) soft corals, (7) sponges, and (8) ascidians^73^. For every deployment the number of cells within each image containing the same habitat type were summed and divided by the total number of cells containing habitat. For simplicity we refer to these data as ‘percent cover’. When grid rectangles were positioned over open water they were classed as ‘no habitat’ and excluded from the overall percent cover and final statistical analyses. If grid rectangles contained habitat, an estimate of relief was also made and categorised from 0 to 5 based on the scheme in Wilson et al. (2007), providing the mean relief for each deployment.

### Environmental and management variables

Water depth (m), GPS coordinate and management status (i.e. whether the stereo-BRUV was deployed in an area open to fishing or a No-Take Area) were recorded for each deployment. The latitude and longitude were also used to calculate the minimum Euclidean distance (m) to the nearest boat ramp which was used as a proxy of relative fishing effort.

### Statistical analysis

We used a generalised additive mixed modelling approach (GAMMs^60^) to explore the relative importance of habitat, management, and environmental variables in determining the abundance and time of arrival of sharks on stereo-BRUVs deployments. We considered five variables in these models: reef cover (continuous), depth (categorical: shallow, deep), distance to closest boat ramp (continuous), standard deviation of relief (continuous), and status (factor: No-Take Area, fishing permitted) (Table 1). Data exploration revealed two distinct clusters in the distribution of the depth of deployments that could not be improved via transformations. Consequently, this parameter was included in the models as a categorical variable as either deep (> 25 m) or shallow (< 25 m). Only the five most abundant species of sharks: *C. amblyrhynchos, T. obesus, C. melanopterus, G. cuvier and C. plumbeus*, were included in this analysis. These were grouped as reef sharks (*C. amblyrhynchos, T. obesus, C. melanopterus*) and apex sharks (*G. cuvier, C. plumbeus*) based on Roff et al. (2016).

Models were initially run separately for each species, but *G. cuvier* and *C. plumbeus* were later summed into an ‘apex shark’ category due to low numbers of sightings. Since the three species of reef sharks (*C. amblyrhynchos, T. obesus, C. melanopterus)* were predominantly recorded at depths < 30 m, we subsetted our data to only include deployments at < 25 m depth (n = 897), to allow for the inclusion of depth as a continuous variable in these models.

We employed a full-subsets generalised additive mixed modelling approach (*FSSgam*^61^) to investigate the relative importance of each variable in driving the abundance of sharks on stereo-BRUV deployments. Given that the occurrence of sharks on stereo-BRUV deployments was highly zero-inflated (~ 90% zeros), a hurdle model approach was used to model shark abundance. This class of model accounts for an excess of zeros through a two-step model structure^62^. The first step fits a binomial probability model to determine whether a zero or non-zero outcome occurs and the second step fits a separate model for positive outcomes. In this analysis, the occurrence of a shark (presence/absence) was modelled across all deployments using a binomial distribution. The next step only included deployments where sharks were present to model the abundance (MaxN > 0) of sharks using a Gamma distribution with a log link. The time of first arrival of each species of shark in the video from each stereo-BRUV deployment was modelled using a Tweedie distribution^63^ and the likelihood of feeding for each species was modelled using a binomial distribution (1 = fed, 0 = did not feed). For each model, the mean latitude and the year of each stereo-BRUV campaign were included as random effects.

Models were fitted using the gam() function in the R package mgcv^64^. Candidate model sets were constructed using the FSSgam package and compared using Akaike’s Information Criterion for small sample size (AICc) and AICc weight values (ω)^65^. To avoid issues with collinearity, only predictors with absolute Pearson correlations coefficient < 0.28 were included in a single candidate model. To ensure models remained ecologically interpretable, only models with up to three predictors were included in any one candidate model. To reduce overfitting and to further ensure that models remained ecologically interpretable, smoothing terms were fit using a cubic regression spline, with the ‘k’ argument limited to 3. The summed AICc weights were used to derive measures of variable importance of each predictor across all candidate models^66^, where higher summed values represent increased importance of that predictor to the response variable. Since models within two AICc units show weak support for one over the other, the most parsimonious model was the model that included the fewest variables and lowest estimated degrees of freedom within two units of the AICc^65^. The variables that were included in the most parsimonious models were plotted to visualise the shape and direction of the predictor variables and the response variables.

## Ethics statement

The research was approved by the University of Western Australia ethics committee (RA/3/100/1317).

## Supplementary Information

Supplementary Information.
