# Supplementary material for: Drivers of variation in occurrence, abundance, and behaviour of sharks on coral reefs
Source: Sci Rep. 2022 Jan 14;12:728. doi: 10.1038/s41598-021-04024-x (PMC8760336; doi:10.1038/s41598-021-04024-x)
Supplement: Supplementary file 1 — Supplementary Information. [file 41598_2021_4024_MOESM1_ESM.docx]

**Drivers of variation in occurrence, abundance, and behaviour of sharks on coral reefs**

**Electronic supplementary material**

Lester E^1,2^, Langlois T^1^, Lindgren I^1^, Birt M^1^, Bond T^1,2^, McLean D^2^, Vaughan B^2^, Holmes T H^3^, Meekan M^2^

^1^The University of Western Australia Oceans Institute and School of Biological Sciences, University of Western Australia, 35 Stirling Highway, Crawley, WA 6009, Australia

^2^Australian Institute of Marine Science, University of Western Australia, 35 Stirling Highway, Crawley, WA 6009, Australia

^3^Department of Biodiversity, Conservation and Attractions, 17 Dick Perry Avenue, Kensington, WA, 6151

Corresponding author: Emily Lester ([Emily.lester@research.uwa.edu.au](mailto:Emily.lester@research.uwa.edu.au))

Table S‑1 Deployment locations and depths of Baited Remote Underwater Stereo-Video Stations (Stereo-BRUVS) campaigns in north-western Australia. Data were collected at Ningaloo Reef, the Montebello Islands and Dampier Archipelago.

| **Campaign** | **Year** |  | **No. deployments** | **Min depth (m)** | | **Max depth (m)** | **Min latitude (°)** | **Max latitude (°)** | **Min longitude (°)** | **Max longitude (°)** |
| --- | --- | --- | --- | --- | --- | --- | --- | --- | --- | --- |
| 1 | 2015 |  | 123 | | 19 | 56.2 | -23.52 | -21.86 | 113.59 | 113.96 |
| 2 | 2015 |  | 169 | | 0.7 | 7.5 | -23.34 | -21.90 | 113.63 | 113.95 |
| 3 | 2015 |  | 114 | | 5.4 | 13.4 | -23.52 | -21.89 | 113.62 | 113.95 |
| 4 | 2014 |  | 272 | | 6.6 | 21.4 | -21.92 | -20.35 | 114.10 | 116.94 |
| 5 | 2014 |  | 136 | | 43 | 59.5 | -21.69 | -20.17 | 114.11 | 115.53 |
| 6 | 2014 |  | 112 | | 7.4 | 12 | -23.50 | -22.03 | 113.62 | 113.89 |
| 7 | 2015 |  | 120 | | 11.4 | 64 | -20.46 | -20.15 | 115.22 | 115.66 |
| 8 | 2015 |  | 79 | | 1.1 | 11.8 | -20.72 | -20.46 | 116.38 | 116.67 |
| 9 | 2015 |  | 69 | | 21.9 | 56.6 | -20.46 | -20.04 | 116.32 | 116.56 |
| 10 | 2015 |  | 156 | | 2 | 10.5 | -22.46 | -21.84 | 114.13 | 114.51 |
| 11 | 2015 |  | 92 | | 1.9 | 12.5 | -21.63 | -21.32 | 114.65 | 115.23 |
